# A Prospective Study on ^18^F-DCFPyL PSMA PET/CT Imaging in Biochemical Recurrence of Prostate Cancer

**DOI:** 10.2967/jnumed.119.226381

**Published:** 2019-11

**Authors:** Etienne Rousseau, Don Wilson, Frédéric Lacroix-Poisson, Andra Krauze, Kim Chi, Martin Gleave, Michael McKenzie, Scott Tyldesley, S. Larry Goldenberg, François Bénard

**Affiliations:** 1 BC Cancer, Vancouver, British Columbia, Canada; 2 Department of Radiology, University of British Columbia, Vancouver, British Columbia, Canada; and; 3 Department of Urologic Sciences, University of British Columbia, Vancouver, British Columbia, Canada

**Keywords:** prostate cancer, prostate specific membrane antigen, biochemical recurrence

## Abstract

^18^F-DCFPyL (2-(3-{1-carboxy-5-[(6-^18^F-fluoro-pyridine-3-carbonyl)-amino]-pentyl}-ureido)-pentanedioic acid), a prostate-specific membrane antigen–targeting radiotracer, has shown promise as a prostate cancer imaging radiotracer. We evaluated the safety, sensitivity, and impact on patient management of ^18^F-DCFPyL in the setting of biochemical recurrence of prostate cancer. **Methods:** Subjects with prostate cancer and biochemical recurrence after radical prostatectomy or curative-intent radiotherapy were included in this prospective study. The subjects underwent ^18^F-DCFPyL PET/CT imaging. The localization and number of lesions were recorded. The uptake characteristics of the 5 most active lesions were measured. A pre- and posttest questionnaire was sent to treating physicians to assess the impact on management. **Results:** One hundred thirty subjects were evaluated. ^18^F-DCFPyL PET/CT localized recurrent prostate cancer in 60% of cases with a prostate-specific antigen (PSA) level of ≥0.4 to <0.5, 78% with a level of ≥0.5 to <1.0, 72% with a level of ≥1.0 to <2.0, and 92% with a level of ≥2.0. Many subjects had few lesions (1 lesion in 40.8%, 2 in 8.5%, and 3 in 4.6%). The number of lesions was significantly related to PSA by ANOVA, but there was a large overlap in the PSA values for number of lesion categories. Total lesion uptake was also significantly related to PSA level. A change in treatment intent occurred in 65.5% of subjects, disease stage changed in 65.5%, and management plans changed in 87.3%. Twenty-two subjects reported mild adverse events after the scan; all resolved completely. **Conclusion:** ^18^F-DCFPyL PET/CT is safe and sensitive for the localization of biochemical recurrence of prostate cancer. This test improved decision making for referring oncologists and changed management for most subjects.

Prostate cancer (PC) is the most prevalent cancer in men in Canada and is the cause of one third of cancer deaths in that population (1). Although biochemical recurrence after therapy can be identified with the prostate-specific antigen (PSA) test, localization of recurrence can be challenging with conventional imaging modalities that cannot match the sensitivity of this blood test (2,3). Precise localization of sites of recurrence is important, as there are options available to treat localized or oligometastatic disease (4,5).

With a new class of PET radiopharmaceuticals targeting the prostate-specific membrane antigen (PSMA), it has become feasible to detect recurrent or metastatic prostate cancer that is otherwise occult on conventional imaging modalities (6–9). ^18^F-DCFPyL (2-(3-{1-carboxy-5-[(6-^18^F-fluoro-pyridine-3-carbonyl)-amino]-pentyl}-ureido)-pentanedioic acid), a radiotracer based on the glutamate-ureido-lysine motif, has the advantage of the longer 110-min half-life of ^18^F compared with ^68^Ga and of ease of regional distribution; it has been used successfully for detection of PSMA-expressing prostate cancer lesions (10–12).

In this study, we aimed to determine the proportion and characteristics of participants with biochemical recurrence who present with limited-extent disease (localized or oligometastatic) that would potentially be amenable to surgical resection or localized irradiation. We also aimed to assess the clinical impact of ^18^F-DCFPyL PET/CT in patient management and to evaluate the safety of this radiopharmaceutical for clinical use.

## MATERIALS AND METHODS

### Ethical Approval

The trial was conducted in compliance with the protocol and with good-clinical-practice guidelines as set out by Health Canada and the institutional Research Ethics Board. The study was approved by the Research Ethics Board of University of British Columbia/BC Cancer and by Health Canada. All procedures performed in studies involving human participants were in accordance with the ethical standards of the institutional or national research committee and with the 1964 Helsinki declaration and its later amendments or comparable ethical standards. Written informed consent was obtained from all participants included in the study.

### Selection of Subjects

Participants with any of the following criteria were enrolled: known prostate cancer with biochemical recurrence after initial curative therapy with radical prostatectomy, with a PSA level higher than 0.4 ng/mL and an additional measurement showing increase; known prostate cancer with biochemical recurrence after initial curative therapy with radiation therapy, with a PSA level higher than 2 ng/mL above the nadir after therapy; castration-resistant prostate cancer with a PSA level of at least 2.0 ng/mL with 2 consecutive rises above the nadir and castrate levels of testosterone (<1.7 nm/L); and participants with findings on other examinations (such as plain x-ray, CT, MRI, or bone scintigraphy) that are suggestive of metastatic disease but not conclusive. Participants were excluded if medically unstable, unable to lie supine for imaging, unable to provide written consent, exceeding the safe weight of the PET/CT bed (204.5 kg) or unable to fit through the PET/CT bore (70-cm diameter), or having an Eastern Cooperative Oncology Group score of more than 2. No treatment was discontinued before the ^18^F-DCFPyL scan.

This is an interim analysis of the first 208 participants of an investigator-initiated clinical trial (clinicaltrials.gov NCT02899312). Only participants meeting the first 2 inclusion criteria were analyzed for this paper (130/208), but all 208 are included in the safety analysis. Repeat scans in the same subjects were not included.

### Study Procedures

Patient demographics were recorded, along with relevant oncologic history, laboratory values, and tumor pathology data. Referring physicians completed a questionnaire describing the intended course of treatment before the ^18^F-DCFPyL PET/CT scan. Participants were followed up 24 h after radiotracer administration to identify adverse events. A second questionnaire was sent to referring physicians a few weeks after the scan to assess changes in management.

^18^F-DCFPyL was synthesized according to a previously published method (13). The administered activity was scaled by body weight (range, 237–474 MBq), allowing a 10% variation in target activity. After a 4-h fast, participants were injected intravenously with ^18^F-DCFPyL. Vital signs were measured before injection, 5–15 min afterward, and after the uptake phase. The subjects could eat between the radiotracer injection and the scan. After a 120-min uptake period, patients were imaged from top of head to mid thigh on a Discovery PET/CT 600 or 690 (GE Healthcare). A CT scan for localization and attenuation correction (120 kV, automatic milliamperage selection [range, 30–200 mA], and noise index of 20) was acquired. PET data were acquired immediately after the CT data over 2–4 min/bed position, adjusted for participant girth, and reconstructed with ordered-subset expectation maximization and point-spread-function modeling.

### Qualitative Image Analysis

Images were interpreted by experienced nuclear medicine physicians on an Oasis (Segami) or AW workstation (GE Healthcare). Physicians completed a qualitative interpretation case report form recording the number of positive lesions (0, 1, 2, 3, 4, 5, 6–10, or >10) and the site of recurrence (local, regional nodes, distant nodes, bone, liver, lung, or other). Regional nodes were considered pelvic, hypogastric, obturator, iliac (internal or external), or sacral; other nodal locations were considered distant. Physicians had access to all clinical data; they recorded scans as positive or negative and rated their confidence in the diagnosis for a total of 6 possible qualitative results (negative: high, moderate, or low; positive: high, moderate, or low).

### Quantitative Image Analysis

Quantitative data were extracted on an AW workstation by a nuclear medicine physician, on images reconstructed without the time-of-flight option for consistency between the 2 scanners. The mean and SD of cardiac blood-pool activity in a 3-cm spheric volume of interest in the left ventricle were recorded as SUV and lean body mass SUV (SUL). Peak and SUV_max_/SUL as well as total lesion uptake for the 5 most active lesions of each scan were recorded using manually corrected semiautomatic contours.

### Statistical Analysis and Computations

Analysis was exploratory. Statistics were computed in R, version 3.5.1 (R Foundation for Statistical Computing). Descriptive statistics included mean, SD, or proportions, as appropriate. Vital signs were analyzed using a mixed-effects model (paired data). PSA doubling time was calculated by fitting to a linear model with logarithmic transformation. Negative doubling times due to treatment effects were excluded from calculation. Subjects were not excluded from the study on the basis of missing data; rather, for each variable or multivariate analysis, the maximum number of evaluable subjects (who had all required variables) was used and reported. Continuous distributions were compared with the Welch *t* test. When analyzing the effect of categoric variables against another categoric variable, the Pearson χ^2^ test was used with *P* values estimated by Monte Carlo simulation (10^6^ repetitions). When the effect of categoric variables was assessed against a continuous variable, a linear model with ANOVA was used. SUV_max_/SUL_max_ dispersion was assessed by coefficient of variation. Statistical significance was defined as a *P* value less than or equal to 0.05.

## RESULTS

### Demographic Characteristics

One hundred thirty subjects were included in the analysis, with demographic parameters reported in Table 1 and Supplemental Table 1 (supplemental materials are available at http://jnm.snmjournals.org). There were 94 subjects (72.3%) with biochemical recurrence after radical prostatectomy and 37 (28.5%) with biochemical recurrence after radiation therapy. Prior treatments included surgery (72.3% of cases), radiotherapy (34.6%), androgen-deprivation therapy (47.7%), or chemotherapy (0.8%), with some participants having received more than one type of therapy. Forty-five subjects received one or more types of radiotherapy: brachytherapy was administered to 27 of 45, external-beam radiotherapy to 20 of 45, intensity-modulated radiation therapy to 4 of 45, and proton therapy to 1 of 45. Overall, the subjects had a mean PSA level of 5.20 ± 6.50 ng/mL with a doubling time of 12.2 ± 11.8 mo (*n* = 113).

**TABLE 1:** Patient Characteristics

	All included		BR after RP only*		BR after RT only*	
Variable	Data	*n*	Data	*n*	Data	*n*
Age (y)	69.1 ± 6.5	130	68.4 ± 6.3	92	70.8 ± 6.9	35
Body weight (kg)	87.4 ± 14.4	130	86.9 ± 14.4	92	87.7 ± 13.5	35
Height (cm)	177.3 ± 6.8	130	176.9 ± 6.8	92	177.5 ± 6.6	35
Injected activity (MBq)	369.2 ± 47.2	130	367.8 ± 47.1	92	371.1 ± 46.0	35
Uptake time (min)	120.4 ± 1.5	130	120.5 ± 1.7	92	120.2 ± 0.6	35
Inclusion criteria^†^						
Known PC after radical prostatectomy with BR	94 (72.3%)	130	92 (100%)	92	0 (0.0%)	35
Known PC after radiation therapy with BR	37 (28.5%)	130	0 (0.0%)	92	35 (100%)	35
PSA at baseline (ng/mL)	5.20 ± 6.50	130	3.03 ± 3.40	92	11.11 ± 8.94	35
PSA doubling time (mo)	12.2 ± 11.8	113	12.0 ± 12.3	78	12.9 ± 11.1	32
Treatment history^†^						
Surgery	94 (72.3%)	130	92 (100%)	92	0 (0.0%)	35
Radiotherapy^†^	45 (34.6%)	130	7 (7.6%)	92	35 (100%)	35
Brachytherapy	27 (60.0%)	45	0 (0.0%)	7	26 (74.3%)	35
External-beam therapy	20 (44.4%)	45	5 (71.4%)	7	13 (37.1%)	35
Intensity-modulated radiation therapy	4 (8.9%)	45	2 (28.6%)	7	2 (5.7%)	35
Proton therapy	1 (2.2%)	45	0 (0.0%)	7	1 (2.9%)	35
^223^RaCl2	0 (0.0%)	45	0 (0.0%)	7	0 (0.0%)	35
Androgen-deprivation therapy	62 (47.7%)	130	39 (42.4%)	92	22 (62.9%)	35
Chemotherapy	1 (0.8%)	130	1 (1.1%)	92	0 (0.0%)	35

* Inclusion criteria.

† Categories are not mutually exclusive.

PC = prostate cancer; BR= biochemical recurrence; RP = radical prostatectomy; RT = radiation therapy.

Data are mean ± SD or proportions.

### Initial Tumor Characteristics

The distribution of Gleason scores was skewed toward intermediate to high grades (6: 13.2%, 3 + 4 = 7: 21.7%, 4 + 3 = 7: 28.7%, 8: 10.1%, 9: 25.6%, 10: 0.8%; *n* = 129). Most had an advanced pathologic T stage, with pT3 and pT4 representing 59.4% (*n* = 64) (Supplemental Table 2).

### Clinical Assessment of PET/CT Scans

**FIGURE 1.**
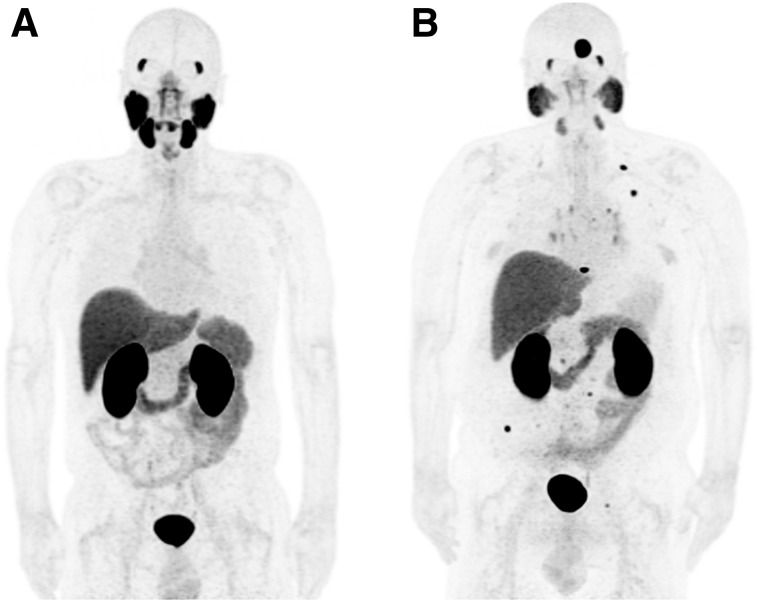
^18^F-DCFPyL PET maximum-intensity projections representative of tracer distribution. (A) Normal biodistribution (significant uptake by lacrimal glands, salivary glands, kidneys, liver, spleen, bowel, and bladder content). (B) Metastatic prostate cancer.

Representative ^18^F-DCFPyL PET/CT scans are shown in Figure 1; 84.6% were positive, with varying certainty levels (81.5% high, 13.1% moderate, and 5.4% low), showing that the readers had good confidence in their findings (Table 2; Supplemental Table 3). A high proportion of participants (53.9%) had only 3 lesions or fewer (1 lesion detected in 40.8%, 2 in 8.5%, and 3 in 4.6%).

**TABLE 2:** Qualitative Assessment of Scans

	All included		BR after RP only*		BR after RT only*	
Variable	Data	*n*	Data	*n*	Data	*n*
Number of lesions		130		92		35
0	20 (15.4%)		19 (20.7%)		0 (0.0%)	
1	53 (40.8%)		35 (38.0%)		18 (51.4%)	
2	11 (8.5%)		6 (6.5%)		5 (14.3%)	
3	6 (4.6%)		6 (6.5%)		0 (0.0%)	
4	3 (2.3%)		3 (3.3%)		0 (0.0%)	
5	7 (5.4%)		5 (5.4%)		2 (5.7%)	
6–10	14 (10.8%)		10 (10.9%)		3 (8.6%)	
>10	16 (12.3%)		8 (8.7%)		7 (20.0%)	
Sites of relapse^†^		130		92		35
Local	35 (26.9%)		13 (14.1%)		22 (62.9%)	
Regional nodes	57 (43.8%)		41 (44.6%)		14 (40.0%)	
Distant nodes	32 (24.6%)		21 (22.8%)		10 (28.6%)	
Bone	26 (20.0%)		20 (21.7%)		6 (17.1%)	
Lung	3 (2.3%)		2 (2.2%)		1 (2.9%)	
Liver	0 (0.0%)		0 (0.0%)		0 (0.0%)	
Other	1 (0.8%)		1 (1.1%)		0 (0.0%)	
Diagnosis		130		92		35
Positive	110 (84.6%)		73 (79.3%)		35 (100%)	
Negative	20 (15.4%)		19 (20.7%)		0 (0.0%)	
Certainty of diagnosis		130		92		35
High	106 (81.5%)		73 (79.3%)		31 (88.6%)	
Moderate	17 (13.1%)		14 (15.2%)		3 (8.6%)	
Low	7 (5.4%)		5 (5.4%)		1 (2.9%)	

* Inclusion criteria.

† Categories are not mutually exclusive.

BR = biochemical recurrence; RP = radical prostatectomy; RT = radiation therapy.

Data are proportions.

In an ANOVA of a linear model in which PSA was analyzed with the number of lesions and Gleason score factors, the number of lesions had a significant effect (*P* < 0.01). However, there was substantial overlap in PSA values for differing numbers of lesions. The initial Gleason score was not significantly associated with PSA. To evaluate for a potential association between PSA value and lesion localization, Gleason score, or number of lesions, participants with disease in only 1 area were selected (*n* = 75). ANOVA of a linear model of PSA against lesion localization, Gleason score, and number of lesions was computed. In that subgroup, no significant association was found. Also, there was substantial overlap in PSA values when plotted against those factors (Supplemental Figs. 1–3). The Gleason score was not related to the number of lesions when evaluated by χ^2^, but there was lack of independence when evaluated against sites of relapse (χ^2^; *P* < 0.01).

**FIGURE 2.**
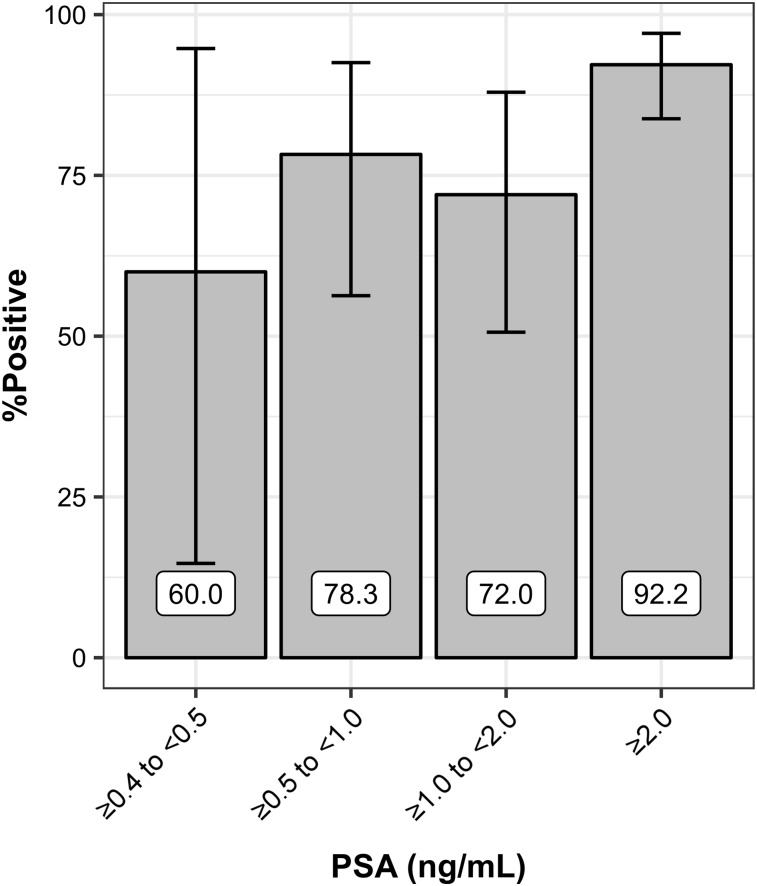
Proportion of positive scans based on PSA level. Error bars represent 95% confidence intervals.

The proportion of positive scans increased with PSA level (Fig. 2; Supplemental Table 4). The PSA values for positive scans (5.80 ± 6.87 ng/mL) were significantly different (Welch *t* test; *P* < 0.001) from those for negative scans (1.86 ± 1.62 ng/mL); however, there was a large overlap in PSA values across those 2 categories.

Active disease was most often identified in regional nodes (43.9%), followed by prostate bed/seminal glands (26.9%), distant nodes (24.6%), bone (20.0%), lung (2.3%), and other sites (0.8%); no liver lesions were identified (Supplemental Fig. 4). Several participants had disease in more than one site. Previous treatments had an influence on lesion distribution, which differed, notably, between patients who previously had surgery with or without androgen-deprivation therapy and those who had radiotherapy with or without androgen-deprivation therapy (χ^2^ test; *P* < 0.01) (Supplemental Fig. 5). In the subset of participants treated with radiotherapy with or without androgen-deprivation therapy, there was trend toward a differing distribution of lesion localization between brachytherapy and external-beam radiotherapy (χ^2^ test; *P* = 0.051); this distribution was calculated while excluding subjects who had had multiple radiotherapy types.

### Evaluation of Lesions

Background uptake was low (SUV_mean_, 1.22 ± 0.22) in the cardiac blood pool. The distribution of lesion uptake had an SUV_max_ range of 1.15–85.04 (mean, 12.43 ± 12.34). SUV_peak_ yielded distributions with a smaller range, 0.86–61.2 (mean, 7.60 ± 7.98). Coefficients of variation of SUL_max_ (97%) and SUL_peak_ (106%) were comparable to those of SUV_max_ (99%) and SUV_peak_ (105%) (Supplemental Table 5). When selecting patients who had 5 or fewer lesions (the maximum recorded on the quantitative assessment), there was a significant relationship between PSA and sum of total lesion uptake (*P* < 0.05) when assessed by ANOVA of a linear model that also accounted for the Gleason score (which also had a significant association with PSA in this reduced data set; *P* < 0.01). Lesion SUV_max_ and SUL_max_ were significantly related to the initial Gleason score when evaluated by a linear model (*P* < 0.05).

### Adverse Events

Vital signs varied at different time points: blood pressure changed from 142 ± 19/82 ± 13 to 146 ± 19/80 ± 9 mm Hg between preinjection values and immediately before the scan. Heart rate changed from 65 ± 14 to 75 ± 16 bpm, and pulse oximetry from 97.6% ± 2.1% to 97.6% ± 2.6%. Those values were statistically significant (except for pulse oximetry) but not considered clinically significant. There were no adverse events during scans. In total, 22 subjects reported mild adverse events after the scan; all resolved completely (Supplemental Table 6).

### Changes in Management

Currently, referring physicians have completed postscan assessments of changes in management for 55 of 130 subjects (Table 3; Supplemental Table 7). A change in treatment intent occurred in 65.5% of subjects, with half of those being directed to palliative care and the other half to curative treatment. Disease stage changed in 65.5% of cases (97.1% of which were upstaged). Findings on ^18^F-DCFPyL scans prompted additional imaging in 23.6% of cases, changed plans for surgery or biopsy in 25.5%, changed plans for systemic therapy in 56.4%, and changed plans for radiotherapy in 47.3%. Physicians indicated that imaging results improved decision making in 89.1% and changed management plans in 87.3%.

**TABLE 3:** Changes in Treatment Intent, Disease Stage, Investigation, Decision Making, or Management Plan

	All included		BR after RP only*		BR after RT only*	
Variable	Data	*n*	Data	*n*	Data	*n*
Change in treatment intent	36 (65.5%)	55	21 (56.8%)	37	13 (86.7%)	15
To palliative	18 (50.0%)	36	10 (47.6%)	21	6 (46.2%)	13
To curative	18 (50.0%)	36	11 (52.4%)	21	7 (53.8%)	13
Change in disease stage	36 (65.5%)	55	24 (64.9%)	37	10 (66.7%)	15
Upstaged	34 (97.1%)	35	23 (100%)	23	9 (90.0%)	10
Downstaged	1 (2.9%)	35	0 (0.0%)	23	1 (10.0%)	10
Ordering of additional diagnostic studies^†^	13 (23.6%)	55	6 (16.2%)	37	7 (46.7%)	15
CT	4 (30.8%)	13	2 (33.3%)	6	2 (28.6%)	7
MRI	5 (38.5%)	13	3 (50.0%)	6	2 (28.6%)	7
Nuclear medicine	1 (7.7%)	13	1 (16.7%)	6	0 (0.0%)	7
Ultrasound	0 (0.0%)	13	0 (0.0%)	6	0 (0.0%)	7
Biopsy	4 (30.8%)	13	0 (0.0%)	6	4 (57.1%)	7
Other^‡^	1 (7.7%)	13	0 (0.0%)	6	1 (14.3%)	7
Imaging results changed plans for surgery or biopsy	14 (25.5%); NA 13 (23.6%)	55	6 (16.2%); NA 10 (27.0%)	37	8 (53.3%); NA 1 (6.7%)	15
Surgery or biopsy added	9 (64.3%)	14	4 (66.7%)	6	5 (62.5%)	8
Surgery or biopsy cancelled	5 (35.7%)	14	2 (33.3%)	6	3 (37.5%)	8
Other	0 (0.0%)	14	0 (0.0%)	6	0 (0.0%)	8
Imaging results changed plans for systemic therapy	31 (56.4%); NA 3 (5.5%)	55	20 (54.1%); NA 2 (5.4%)	37	9 (60.0%); NA 1 (6.7%)	15
Systemic therapy started	23 (74.2%)	31	15 (75.0%)	20	6 (66.7%)	9
Systemic therapy not initiated/cancelled	8 (25.8%)	31	5 (25.0%)	20	3 (33.3%)	9
Systemic therapy changed	0 (0.0%)	31	0 (0.0%)	20	0 (0.0%)	9
Imaging results changed plans for radiotherapy	26 (47.3%); NA 9 (16.4%)	55	22 (59.5%); NA 6 (16.2%)	37	4 (26.7%); NA 1 (6.7%)	15
Radiotherapy added	13 (52.0%)	25	11 (52.4%)	21	2 (50.0%)	4
Radiotherapy cancelled	9 (36.0%)	25	8 (38.1%)	21	1 (25.0%)	4
Radiotherapy prescription changed	3 (12.0%)	25	2 (9.5%)	21	1 (25.0%)	4
Imaging results improved decision making	49 (89.1%)	55	33 (89.2%)	37	14 (93.3%)	15
Imaging results changed subject’s management plan	48 (87.3%)	55	32 (86.5%)	37	14 (93.3%)	15

* Inclusion criteria.

† Categories are not mutually exclusive.

‡ Repeat PET a few months after start of androgen-deprivation therapy.

BR = biochemical recurrence; RP = radical prostatectomy; RT = radiation therapy; NA = not applicable.

Data are proportions.

## DISCUSSION

This study aimed to determine the sensitivity and safety of ^18^F-DCFPyL PET/CT for the detection of prostate cancer relapse in the context of biochemical recurrence. Since the initial publication by Rowe et al. in 2015 on 9 patients, several small studies have been published on this tracer for prostate cancer, many of them by the same groups (8,10–12,14–20). This interim analysis evaluated a large prospective cohort of subjects who participated in an investigator-initiated ^18^F-DCFPyL PET/CT imaging study in Vancouver, Canada.

Although the definition of oligometastatic disease in prostate cancer is still evolving, many participants had a low number of lesions that would fall under this category (53.9% had 1–3 lesions) (21–23). Although more research is needed to assess its efficacy, there is a potential for localized therapy (i.e., resection or stereotactic body radiation therapy) with minimal risk of serious adverse events (23,24). In this setting, ^18^F-DCFPyL PET/CT may be useful to identify disease occult on other imaging modalities that could be amenable to more aggressive treatment (25). Furthermore, in 65.4% of participants, disease was located in regional nodes or presented as local recurrence. For subjects who were treated surgically, this disease would potentially be amenable to salvage pelvic irradiation.

Although the number of lesions reported on imaging was significantly related to PSA values at baseline, there was an important overlap in PSA range between groupings based on the number of lesions. Such an overlap is to be expected, as the number of lesions is not a good indicator of tumor burden because of size variations. Conversely, there was a significant relation between total lesion uptake and PSA. However, no PSA value was predictive of oligometastatic disease in this population.

Compared with the detection rates presented by Eiber for ^68^Ga-PSMA HBED-CC—57.9%, 72.7%, 93%, and 96.8%, with respective PSA intervals of 0.2–0.5, 0.5–1.0, 1.0–2.0, and ≥2.0 ng/mL—our study achieved similar results, with detection rates of 60% ± 80% (exact 95% confidence interval), 78% ± 36%, 72% ± 37%, and 92% ± 14% at equivalent intervals (≥0.4 to <0.5, ≥0.5 to <1.0, ≥1.0 to <2.0, and ≥2.0, respectively) (26). The lower detection rate in the 1.0–2.0 interval for ^18^F-DCFPyL may be attributable to random variations and remains within the 95% confidence interval for the proportion. This is also similar to other ^68^Ga-PSMA studies reported in a review by Evans et al. and to detection rates reported for ^18^F-PSMA-1007 (61.5%, 74.5%, 90.1%, and 94.1%) (27,28). ^18^F-DCFPyL, in the context of the inclusion criteria of the present analysis, appears to have an overall similar sensitivity to other radiotracers.

The distribution of active disease was dependent on prior therapy. There was a greater proportion of local recurrence after radiotherapy than after surgery. This study was not designed to evaluate primary treatment modalities. Referral patterns for inclusion into the study might account for some of these differences.

Change in treatment intent occurred in 65.5% of subjects, all of whom had a change in disease stage. In comparison with ^68^Ga-PSMA-11, Afaq et al. reported changes in management plans in 39% of patients and Hope et al. in 59.6%. Koerber et al. reported changes in radiotherapeutic management of 56.3% in patients with PSA persistence after surgery or recurrence after definitive therapy (29–31). A systematic review by Han et al. reported a change in management in 54% (95% confidence interval, 47%–60%) (32). PSA at baseline was determined not to be a significant factor for change in management, treatment intent, or disease stage or for ordering additional diagnostic studies when assessed by logit analysis. In the metaanalysis by Han et al., the metaregression had not shown PSA to be a significant factor for change in management either, but there was a tendency toward a greater proportion of management changes in studies with greater PSA levels before PET (32).

Although a small proportion of participants reported undesirable events, all were mild and resolved completely. There was no serious adverse event. Our results indicate that ^18^F-DCFPyL can be considered safe for injection in humans (10,11).

As a limitation to this study, the fact that not all referring physicians (55/130) had completed the questionnaire for change in management at the time of analysis could reflect a reporting bias in favor of helpful scans.

## CONCLUSION

^18^F-DCFPyL PET/CT imaging identified sites of recurrent prostate cancer in most subjects and was well tolerated, with no serious adverse events. A large proportion of subjects meeting the inclusion criteria for this analysis had 3 or fewer lesions identified on the scan. ^18^F-DCFPyL PET/CT imaging improved decision making for referring oncologists and changed management plans for most subjects.

## DISCLOSURE

Financial support was received from the Canadian Institutes of Health Research (FDN-148465), BC Cancer Foundation, BC Leading Edge Endowment Fund, and Terry Fox Research Institute. No other potential conflict of interest relevant to this article was reported.

## KEY POINTS

**QUESTION:** What is the impact of ^18^F-DCFPyL PET/CT on patient management in the setting of biochemical recurrence of prostate cancer?
**PERTINENT FINDINGS:** In this analysis of a prospective clinical trial, ^18^F-DCFPyl changed the management plan for 87.3% of patients and the disease stage for 65.5%, with no serious adverse events.
**IMPLICATIONS FOR PATIENT CARE:** ^18^F-DCFPyL PET/CT is safe and changed the management for most subjects.
