# Supplementary material for: A Prospective Study on 18F-DCFPyL PSMA PET/CT Imaging in Biochemical Recurrence of Prostate Cancer
Source: J Nucl Med. 2019 Nov;60(11):1587–93. doi: 10.2967/jnumed.119.226381 (PMC6836862; doi:10.2967/jnumed.119.226381)
Supplement: Supplementary file 1 [file jnm226381SupplementalData.pdf]

Supplemental Table 1: Patient characteristics (additional subgroups)

	ADT naive		Prior or ongoing ADT	
**Variable**	**Value**	**n**	**Value**	**n**
Age (years)	69.1±6.3	68	69.0±6.8	62
Body weight (kg)	86.8±15.1	68	88.2±13.6	62
Height (cm)	177.3±6.0	68	177.3±7.6	62
Injected activity (MBq)	366.4±45.6	68	372.4±49.0	62
Uptake time (min)	120.5±1.8	68	120.3±0.9	62
Inclusion criteria†				
Known PC after radical prostatectomy with BR	54 (79.4%)	68	40 (64.5%)	62
Known PC after radiation therapy with BR	14 (20.6%)	68	23 (37.1%)	62
PSA at baseline (ng/mL)	3.55±3.61	68	7.01±8.29	62
PSA doubling time (months)	15.3±13.8	55	9.3±8.7	58
Treatment history†				
Surgery	54 (79.4%)	68	40 (64.5%)	62
Radiotherapy†	17 (25.0%)	68	28 (45.2%)	62
Brachytherapy	13 (76.5%)	17	14 (50.0%)	28
External beam	5 (29.4%)	17	15 (53.6%)	28
IMRT	0 (0.0%)	17	4 (14.3%)	28
Proton	0 (0.0%)	17	1 (3.6%)	28
Radium-223	0 (0.0%)	17	0 (0.0%	28
Other	0 (0.0%)	17	0 (0.0%)	28
ADT	0 (0.0%)	68	62 (100%)	62
Chemotherapy	0 (0.0%)	68	1 (1.6%)	62

PC: prostate cancer; BR: biochemical recurrence; ADT: Androgen deprivation therapy; IMRT: Intensity-modulated radiation therapy. †Categories are not mutually exclusive.

Supplemental Table 2: Staging and Gleason Score. Includes pathological TNM and Gleason score when available.

	All included		BR post RP only		BR post RT only		ADT Naive		Prior or ongoing ADT	
**Variable**	**Value**	**n**	**Value**	**n**	**Value**	**n**	**Value**	**n**	**Value**	**n**
Pathological TNM		64		63		0		38		26
pT2*	0 (0.0%)		0 (0.0%)		0 (0.0%)		0 (0.0%)		0 (0.0%)	
pT2a	2 (3.1%)		2 (3.2%)		0 (0.0%)		0 (0.0%)		2 (7.7%)	
pT2b	3 (4.7%)		3 (4.8%)		0 (0.0%)		1 (2.6%)		2 (7.7%)	
pT2c	21 (32.8%)		21 (33.3%)		0 (0.0%)		14 (36.8%)		7 (26.9%)	
pT3a	11 (17.2%)		11 (17.5%)		0 (0.0%)		7 (18.4%)		4 (15.4%)	
pT3b	27 (42.2%)		26 (41.3%)		0 (0.0%)		16 (42.1%)		11 (42.3%)	
pT4	0 (0.0%)		0 (0.0%)		0 (0.0%)		0 (0.0%)		0 (0.0%)	
pNx	7 (10.9%)		7 (11.1%)		0 (0.0%)		3 (7.9%)		4 (15.4%)	
pN0	46 (71.9%)		46 (73.0%)		0 (0.0%)		29 (76.3%)		17 (65.4%)	
pN1	11 (17.2%)		10 (15.9%)		0 (0.0%)		6 (15.8%)		5 (19.2%)	
Gleason score		129		91		35		67		62
6	17 (13.2%)		8 (8.8%)		8 (22.9%)		8 (11.9%)		9 (14.5%)	
7 (3+4)	28 (21.7%)		17 (18.7%)		11 (31.4%)		19 (28.4%)		9 (14.5%)	
7 (4+3)	37 (28.7%)		27 (29.7%)		9 (25.7%)		19 (28.4%)		18 (29.0%)	
8	13 (10.1%)		12 (13.2%)		1 (2.9%)		7 (10.4%)		6 (9.7%)	
9	33 (25.6%)		26 (28.6%)		6 (17.1%)		13 (19.4%)		20 (32.3%)	
10	1 (0.8%)		1 (1.1%)		0 (0.0%)		1 (1.5%)		0 (0.0%)	

*Data not available to specify a or b stage.

Supplemental Table 3: Qualitative assessment of scans (additional subgroups)

	ADT naive		Prior or ongoing ADT	
**Variable**	**Value**	**n**		
Number of lesions		68		62
0	14 (20.6%)		6 (9.7%)	
1	26 (38.2%)		27 (43.5%)	
2	7 (10.3%)		4 (6.5%)	
3	0 (0.0%)		6 (9.7%)	
4	1 (1.5%)		2 (3.2%)	
5	6 (8.8%)		1 (1.6%)	
6-10	10 (14.7%)		4 (6.5%)	
>10	4 (5.9%)		12 (19.4%)	
Sites of relapse†		68		62
Local	17 (25.0%)		18 (29.0%)	
Regional nodes	33 (48.5%)		24 (38.7%)	
Distant nodes	14 (20.6%)		18 (29.0%)	
Bone	9 (13.2%)		17 (27.4%)	
Lung	1 (1.5%)		2 (3.2%)	
Liver	0 (0.0%)		0 (0.0%)	
Other	1 (1.5%)		0 (0.0%)	
Diagnosis		68		62
Positive	54 (79.4%)		56 (90.3%)	
Negative	14 (20.6%)		6 (9.7%)	
Certainty of diagnosis		68		62
High	52 (76.5%)		54 (87.1%)	
Moderate	11 (16.2%)		6 (9.7%)	
Low	5 (7.4%)		2 (3.2%)	

Supplemental Table 4: Proportion of positive scans based on PSA levels

	All included		BR post RP only		BR post RT only	
**Variable**	**Value**	**n**	**Value**	**n**	**Value**	**n**
Proportion of positive scans						
>= 0.4 to < 0.5	60.0%	5	60.0%	5	-	0
>= 0.5 to < 1.0	78.3%	23	81.8%	22	-	0
>= 1.0 to < 2.0	72.0%	25	72.0%	25	-	0
>= 2.0	92.2%	77	85.0%	40	100%	35
>= 2.0 to < 5.0	84.8%	33	79.2%	24	100.0%	7
>= 5.0 to < 10.0	96.2%	26	90.9%	11	100.0%	15
>= 10.0 to < 15.0	100.0%	11	100.0%	4	100.0%	7
>= 15.0 to < 20.0	100.0%	2	-	0	100.0%	2
>= 20.0 to < 25.0	100.0%	2	100.0%	1	100.0%	1
>= 25.0 to < 30.0	-	0	-	0	-	0
>= 30.0	100.0%	3	-	0	100.0%	3

Supplemental Table 5: Characteristics of the five most active lesions of each scan and blood pool activity.

Variable	SUV	SUL	n
Cardiac blood pool (mean uptake)	1.22±0.22	0.93±0.15	130
Lesion (max uptake)			290
Mean	12.43	9.29	
Minimum	1.15	0.90	
Maximum	85.04	62.39	
Standard deviation	12.34	9.01	
Lesion (peak uptake)			282
Mean	7.60	5.77	
Minimum	0.86	0.69	
Maximum	61.2	48.3	
Standard deviation	7.98	6.11	

Supplemental Table 6: List of adverse events

Adverse event	Severity	Resolved	Related
Tiredness after scan, resolved after sleeping	mild	yes	Unlikely
Tiredness	mild	yes	Not related
Tiredness & diarrhea overnight	mild	yes	Not related
Flu-like symptoms	mild	yes	Unlikely
Tiredness	mild	yes	Unlikely
'Floaters' in right eye	mild	yes	Not related
Headache and tired	mild	yes	Unlikely
Dark red blood blisters on left arm where injection made, no pain or discomfort.	mild	yes	Possibly
Tiredness	mild	yes	Unlikely
Palpitations	mild	yes	Not related
Felt vertigo symptoms	mild	yes	Unlikely
Felt dizzy/nauseous	mild	yes	Possibly
Dizzy/nauseous - for 5 - 10 minutes after leaving the department.	mild	yes	Possibly
Tired	mild	yes	Unlikely
Chest pain*	mild	yes	Possibly
Metallic taste in mouth	mild	yes	Possibly
Dizzy first thing in the morning	mild	yes	Possibly
Tired	mild	yes	Unlikely
Tired and a bit 'worn out', loose stool, no nausea	mild	yes	Unlikely
Diarrhea	mild	yes	Unlikely
Light headed about 1 hour after the injection. Felt better after laying down for 30 minutes on the scanner bed, during the scan.	mild	yes	Unlikely
Headache	mild	yes	Unlikely
Right lower back muscle ache.	mild	yes	Unlikely
Extra tired	mild	yes	Unlikely
Tiredness/slightly dizzy*	mild	yes	Possibly
Low appetite/slight nausea*	mild	yes	Possibly
Arm sore from IV	mild	yes	Probably

* The subjects did not think the symptoms were related to scan.
Some subjects experienced more than one symptom.

Supplemental Table 7: Changes in treatment intent, disease stage, investigation, decision-making or management plan (additional subgroups)

	ADT naive		Prior or ongoing ADT	
**Variable**	**Value**	**n**		
Change in treatment intent	20 (66.7%)	30	16 (64.0%)	25
To Palliative	9 (45.0%)	20	9 (56.3%)	16
To Curative	11 (55.0%)	20	7 (43.8%)	16
Change in disease stage	20 (66.7%)	30	16 (64.0%)	25
Upstaged	18 (94.7%)	19	16 (100%)	16
Downstaged	1 (5.3%)	19	0 (0.0%)	16
Ordering of additional diagnostic studies†	8 (26.7%)	30	5 (20.0%)	25
Computed tomography	2 (25.0%)	8	2 (40.0%)	5
Magnetic resonance imaging	4 (50.0%)	8	1 (20.0%)	5
Nuclear medicine	1 (12.5%)	8	0 (0.0%)	5
Ultrasound	0 (0.0%)	8	0 (0.0%)	5
Biopsy	2 (25%)	8	2 (40.0%)	5
Other*	0 (0.0%)	8	1 (20.0%)	5
Imaging results changed plans for surgery or biopsy	8 (26.7%)<br>NA: 7 (23.3%)	30	6 (24.0%)<br>NA: 6 (24.0%)	25
Surgery or biopsy added	5 (62.5%)	8	4 (66.7%)	6
Surgery or biopsy cancelled	3 (37.5%)	8	2 (33.3%)	6
Other**	0 (0.0%)	8	0 (0.0%)	6
Imaging results changed plans for systemic therapy	14 (46.7%)<br>NA: 2 (6.7%)	30	17 (68.0%)<br>NA: 1 (4.0%)	25
Systemic therapy started	11 (78.6%)	14	12 (70.6%)	17
Systemic therapy not initiated/cancelled	3 (21.4%)	14	5 (29.4%)	17
Systemic therapy changed	0 (0.0%)	14	0 (0.0%)	17
Imaging results changed plans for radiotherapy	12 (40.0%)<br>NA: 5 (16.7%)	30	14 (56.0%)<br>NA: 4 (16.0%)	25
Radiotherapy added	6 (54.6%)	11	7 (50.0%)	14
Radiotherapy cancelled	3 (27.3%)	11	6 (42.9%)	14
Radiotherapy prescription changed	2 (18.2%)	11	1 (7.1%)	14
Imaging results improved decision-making	26 (86.7%)	30	23 (92.0%)	25
Imaging results changed subject's management plan	25 (83.3%)	30	23 (92.0%)	25

Note: one referring physician did not indicate on follow-up the detailed change in disease stage / plans for radiotherapy (awaiting biopsy).

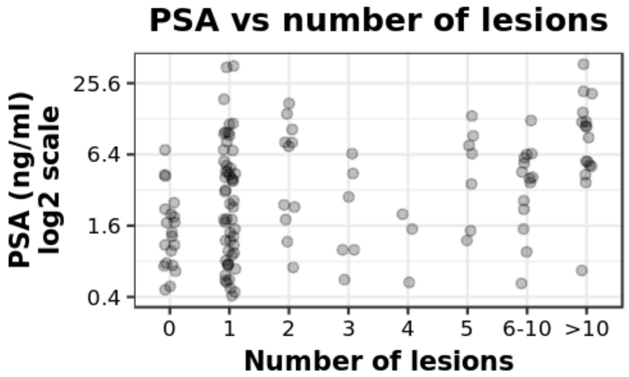

Supplemental Figure 1. PSA vs number of lesions. This represents the PSA values (on a log_2 scale) in each number of lesions category.

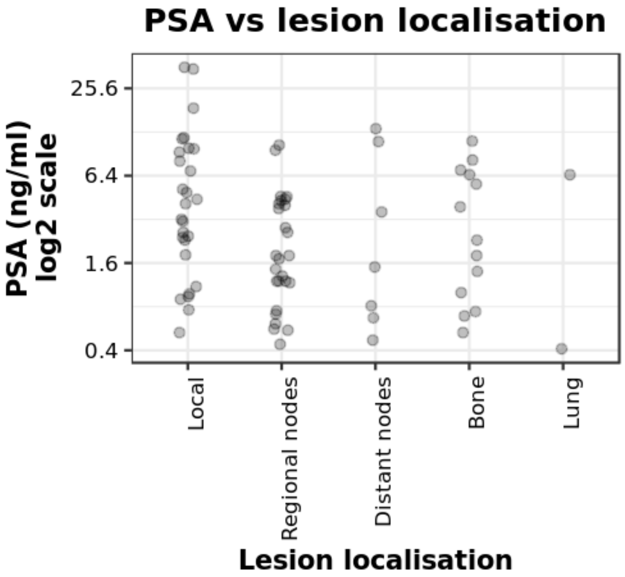

Supplemental Figure 2. PSA vs lesion localisation. Presented lesion localisation are mutually exclusive (i.e. those subjects had lesions in only one localisation).

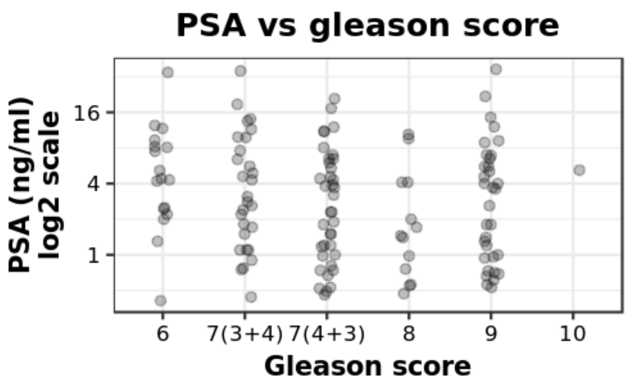

Supplemental Figure 3. PSA vs Gleason score.

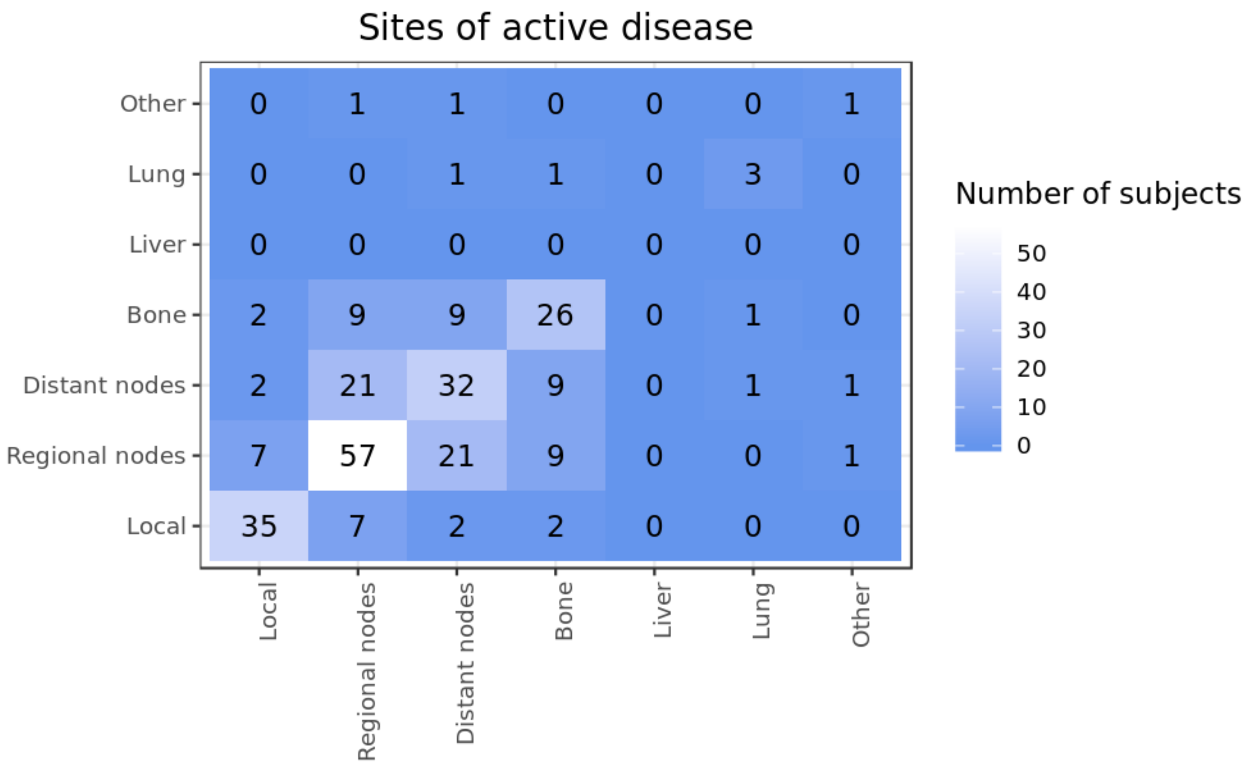

Supplemental Figure 4. Joint histogram of lesion distribution. The graph shows on the diagonal the number of subjects that had disease in the specified location. Each cell shows the number of subjects that had disease simultaneously in those two locations. Categories are not mutually exclusive (a subject may have disease in more than one location, some in more than two).

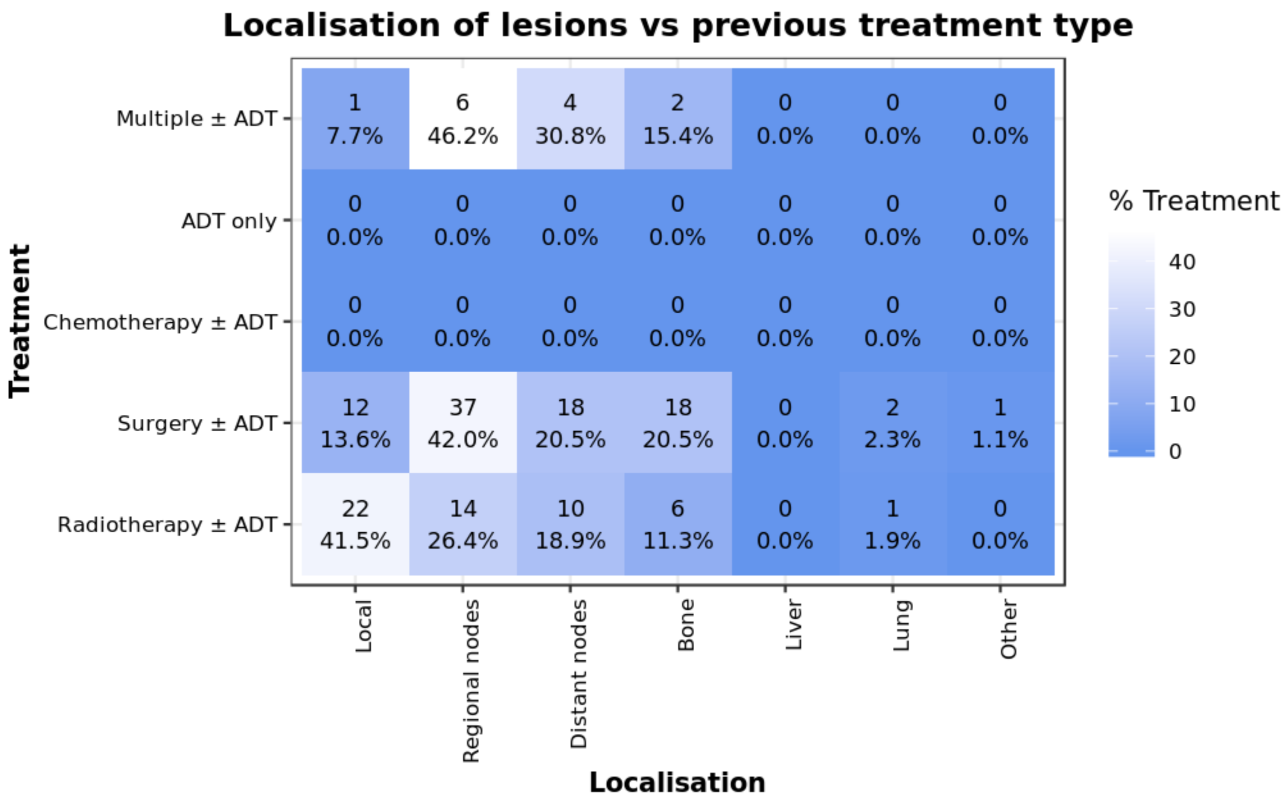

Supplemental Figure 5. Localization of lesions versus previous treatment types. The number in the cells represents the number of subjects that had a recurrence in the region specified on the horizontal axis for each treatment type. A subject may have had recurrence in more than one site. Calculated percentages are cumulative for treatments (calculated by adding cells in each row and dividing by total). When subjects had more than one treatment, they were aggregated in the "Multiple ± ADT" category and excluded from the other categories. ADT: androgen deprivation therapy.

STARD STATEMENT CHECKLIST

**Section & Topic**	**No**	**Item**	**Item included**
Title or abstract			
	1	Identification as a study of diagnostic accuracy using at least one measure of accuracy (such as sensitivity, specificity, predictive values, or AUC)	Yes
Abstract			
	2	Structured summary of study design, methods, results, and conclusions (for specific guidance, see STARD for Abstracts)	Yes
Introduction			
	3	Scientific and clinical background, including the intended use and clinical role of the index test	Yes
	4	Study objectives and hypotheses	Yes
Methods			
Study design	5	Whether data collection was planned before the index test and reference standard were performed (prospective study) or after (retrospective study)	Yes
Participants	6	Eligibility criteria	Yes
	7	On what basis potentially eligible participants were identified (such as symptoms, results from previous tests, inclusion in registry)	Yes
	8	Where and when potentially eligible participants were identified (setting, location and dates)	Yes<br>Study start date: August 3, 2017. Can be derived from clininicaltrial.gov identifier.
	9	Whether participants formed a consecutive, random or convenience series	Yes
Test methods	10a	Index test, in sufficient detail to allow replication	Yes
	10b	Reference standard, in sufficient detail to allow replication	Not applicable<br>(no reference test)
	11	Rationale for choosing the reference standard (if alternatives exist)	Not applicable<br>(no reference test)
	12a	Definition of and rationale for test positivity cut-offs or result categories of the index test, distinguishing pre-specified from exploratory	Yes<br>Cutoffs not applicable<br>Result categories defined in methods.
	12b	Definition of and rationale for test positivity cut-offs or result categories of the reference standard, distinguishing pre-specified from exploratory	Not applicable<br>(no reference test)

	13a	Whether clinical information and reference standard results were available to the performers/readers of the index test	Yes
	13b	Whether clinical information and index test results were available to the assessors of the reference standard	Not applicable (no reference test)
Analysis	14	Methods for estimating or comparing measures of diagnostic accuracy	Yes
	15	How indeterminate index test or reference standard results were handled	Not applicable (no reference test)
	16	How missing data on the index test and reference standard were handled	Yes
	17	Any analyses of variability in diagnostic accuracy, distinguishing pre-specified from exploratory	Yes (analyses were performed on data collected prospectively specified by study protocol).
	18	Intended sample size and how it was determined	Yes. Stated that this is an interim analysis.
Results			
Participants	19	Flow of participants, using a diagram	Described textually.
	20	Baseline demographic and clinical characteristics of participants	Yes
	21a	Distribution of severity of disease in those with the target condition	Yes
	21b	Distribution of alternative diagnoses in those without the target condition	Not applicable (no reference test)
	22	Time interval and any clinical interventions between index test and reference standard	Not applicable (no reference test)
Test results	23	Cross tabulation of the index test results (or their distribution) by the results of the reference standard	Not applicable (no reference test)
	24	Estimates of diagnostic accuracy and their precision (such as 95% confidence intervals)	Yes
	25	Any adverse events from performing the index test or the reference standard	Yes (There were no adverse events)
Discussion			
	26	Study limitations, including sources of potential bias, statistical uncertainty, and generalisability	Yes
	27	Implications for practice, including the intended use and clinical role of the index test	Yes
Other information			
	28	Registration number and name of registry	Yes

	29	Where the full study protocol can be accessed	Yes (information accessible on clinicaltrials.gov)
	30	Sources of funding and other support; role of funders	Yes.
